# Life expectancy without depression increases among Brazilian older adults

**DOI:** 10.1590/S1518-8787.2016050005900

**Published:** 2016-04-15

**Authors:** Flávia Cristina Drumond Andrade, Fan Wu, Maria Lúcia Lebrão, Yeda Aparecida de Oliveira Duarte

**Affiliations:** I Department of Kinesiology and Community HealthCollege of Applied Health SciencesUniversity of Illinois at Urbana-ChampaignChampaignILEUADepartment of Kinesiology and Community Health. College of Applied Health Sciences. University of Illinois at Urbana-Champaign. Champaign, IL, EUA; II San Francisco Department of Public HealthCommunity Behavioral Health ServicesSan FranciscoCAEUA San Francisco Department of Public Health. Community Behavioral Health Services. San Francisco, CA, EUA; III Universidade de São PauloDepartamento de EpidemiologiaFaculdade de Saúde PúblicaUniversidade de São PauloSão PauloSPBrasilDepartamento de Epidemiologia. Faculdade de Saúde Pública. Universidade de São Paulo. São Paulo, SP, Brasil; IV Universidade de São PauloDepartamento de Enfermagem Médico-CirúrgicaEscola de EnfermagemUniversidade de São PauloSão PauloSPBrasilDepartamento de Enfermagem Médico-Cirúrgica. Escola de Enfermagem. Universidade de São Paulo. São Paulo, SP, Brasil

**Keywords:** Aged, Life Expectancy, Aging, Depression, epidemiology, Gender and Health, Health Surveys

## Abstract

**OBJECTIVE:**

To estimate life expectancy with and without depressive symptoms in older adults for the years 2000 and 2010.

**METHODS:**

We evaluated individuals aged 60 years or older (n = 1,862 in 2000 and n = 1,280 in 2010), participants of the *Saúde, Bem-Estar e Envelhecimento* (SABE – Health, Wellbeing and Aging) study in in Sao Paulo, Southeastern Brazil. Depression was measured using the shorter version of the Geriatric Depression Scale (GDS-15); respondents scoring ≥ 6 were classified as having depression. Estimates of life expectancy with and without depression were obtained using the Sullivan method.

**RESULTS:**

Data from 2000 indicate that 60-year-old men could expect to live, on average, 14.7 years without depression and 60-year-old women could expect to live 16.5 years without depression. By 2010, life expectancy without depression had increased to 16.7 years for men and 17.8 years for women. Expected length of life with depression differed by sex, with women expected to live more years with depression than men.

**CONCLUSIONS:**

Between 2000 and 2010, life expectancy without depression in Sao Paulo increased. However, older adults in Brazil, especially older women, still face a serious burden of mental illness.

## RESUMO

**OBJETIVO:**

Estimar a expectativa de vida com e sem sintomas depressivos em idosos para os anos 2000 e 2010.

**MÉTODOS:**

Foram avaliados (n = 1,862 in 2000 e n = 1,280 in 2010) idosos (60 anos ou mais), participantes do estudo Saúde, Bem-Estar e Envelhecimento (SABE), coletado em São Paulo, Brasil. A versão mais curta da escala de depressão geriátrica (GDS-15) foi usada para classificar a depressão. Idosos com pontuação ≥ 6 foram classificados como tendo depressão. As estimativas de expectativa de vida com e sem depressão foram obtidas usando o método de Sullivan.

**RESULTADOS:**

Os dados de 2000 indicaram que, em média, homens e mulheres de 60 anos de idade poderiam esperar viver, respectivamente, 14,7 e 16,5 anos sem depressão. Em 2010, a expectativa de vida sem depressão aumentou para 16,7 anos para homens e 17,8 anos para mulheres. A duração da vida com depressão diferiu por sexo – as mulheres idosas esperam viver mais anos com depressão do que os homens.

**CONCLUSÕES:**

Entre 2000 e 2010, a expectativa de vida sem sintomas depressivos em São Paulo aumentou. Entretanto, os idosos no Brasil, sobretudo as mulheres, ainda enfrentam problemas relacionados às doenças mentais.

## INTRODUCTION

Depression is a common mental health condition among older adults in Brazil. Researchers have estimated that more than 25.0% of community-dwelling older adults in Brazil have clinically significant depressive symptoms and 7.0% have major depression^4^. These rates are even higher among older adults who are hospitalized or living in long-term facilities. A recent meta-analysis found that the prevalence of clinically significant depressive symptoms varied from 20.0%-56.0% among hospitalized older adults in Brazil, and the rates among older adults in long-term care facilities varied from 11.0%-65.0%^11^. Chisholm et al.^12^ estimated that it would cost US$170 *per capita* per year to cover the direct expenses of treating individuals with subclinical depression in the Country.

Experiencing depression at an older age has important implications for individuals, families and society. Among older adults, depression causes individual and family suffering and is associated with poor adherence to recommended medical treatment^15^, poor lifestyle behaviors^34^, cognitive impairment^24^, and physical disability^9^^,^^27^. In turn, these factors are associated with lower quality of life^7^^,^^16^, higher level of health care use^7^^,^^8^, and higher mortality rates^28^.

Many factors can trigger the onset of depressive symptoms among older adults. Medical disorders such as cancer, metabolic disorder, heart disease, and stroke are commonly associated with depressive symptoms^28^. Limitations in daily activities, cognition, and mobility as well as social changes (such as retirement, bereavement, social isolation, and relocation) also influence mood and distress, leading to depressive symptoms. Because the prevalence of these conditions increases with age, researchers and policy makers should not overlook depressive symptoms among the older population.

Life expectancy among older adults in Brazil has increased in recent decades. Life expectancy at birth increased from 51 to 72 years from 1950-2010, and is expected to reach 77.4 years by 2030^a^. During the same period, the proportion of older adults grew from 5.0% of the total population to about 10.0%. By 2030, the older adult population is expected to more than double, reaching 40.7 million people or 17.1% of the total Brazilian population^b^.

While previous studies have provided important estimates of the prevalence of clinically significant depressive symptoms and major depressive symptoms among older adults in Brazil, as well as the factors associated with those conditions, to the best of our knowledge, no studies have estimated the length of life with depressive symptoms, information that would help researchers and policy makers better understand the burden of this condition for this fast-aging society. Given that life expectancy among older adults in Brazil has increased in recent decades, estimates of life expectancy with and without depression are critical to the accurate determination of current and future social and medical service needs.

In this study, we estimated life expectancy with and without depressive symptoms among older adults for the years 2000 and 2010.

## METHODS

This study analyzed data from the *Saúde, Bem-Estar e Envelhecimento* (SABE – Health, Wellbeing and Aging) survey, a longitudinal study of a probabilistic sample of older adults (age 60 and older) of both sexes living in the city of Sao Paulo, SP, Southeastern Brazil. The first wave of the SABE survey was conducted in 2000 with a sample of 2,143 respondents obtained using a two-stage stratified sampling method. Individuals aged 75 or older were oversampled to compensate for the group’s higher mortality rate and smaller probability of being sampled. Details of the study sampling methodology can be found elsewhere^22^. In 2006, a second wave of data was collected (n = 1,413). The sample for the follow-up data collection included the 1,115 older adults from the baseline study and 298 new respondents age 60-64. Data collection procedures were the same as in the baseline study. In 2010, a third wave of data was collected (n = 1,345). Three groups participated in that wave: a) 748 respondents from the baseline study, b) 242 respondents from the 2006 study, and c) 355 new respondents age-64.

The study reported here focuses on the cross-sectional prevalence rates of depression estimated from data obtained in 2000 and 2010. Sample weights for 2000 were calculated based on the 2000 census and weights for the 2010 sample were based on the 2010 census.

Depression was measured using the shorter version of the Geriatric Depression Scale (GDS-15) in the SABE survey. The GDS-15 short form was based on the GDS-30^37^ and has been widely used in clinical and population-based studies as a screening instrument for late-life depression. Several studies have analyzed the scale’s validity and reliability, and have found that the instrument has good specificity and sensitivity^30^. SABE used a GDS-15 questionnaire validated for use in Brazil^1^. Individuals with missing data on more than five items were excluded from the analysis. We used prorated procedures to estimate scores for respondents with missing data on five or fewer items. The Cronbach’s alpha coefficient for the total scale was 0.82 for both years. We used a GDS-15 cutoff score of ≥ 6 points to classify individuals as having depression^1^.

Mortality data were obtained from the *Fundação Sistema Estadual de Análise de Dados* (SEADE Foundation – State System for Data Analysis Foundation)^c^, which provides data on social, demographic, and economic status for the State of Sao Paulo. We generated estimates for 2000 and 2010 using the 1999-2000-2001 and 2009-2010-2011 tables.

Sex was included as a covariate when disaggregating the data, given sex differences in mortality and prevalence of depressive symptoms. We estimated prevalence rates and 95% confidence intervals using sampling weights with Stata-SE version 12.1. Logistic regressions were used to assess sex and wave-related differences.

The Sullivan method (Sullivan, 1997) was used to estimate life expectancy with and without depression. The method employs a standard life table with two states, alive and dead. The alive state is subdivided into two categories, healthy (nondepressed) and unhealthy (depressed), using data on the observed prevalence of depression^6^^,^^22^^,^^d^. The major inputs are the age-specific prevalence of depressed and nondepressed states in the population and age-specific mortality rates. The expected years in the nondepressed state and expected years in the depressed state were estimated by applying the age- and sex-specific cross-sectional prevalence rates for nondepressed and depressed respondents, respectively, to the person-years lived in different age groups (derived from the period life tables).

Healthy Life Expectancy (HLE)
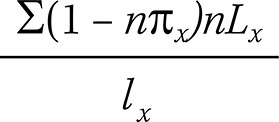


and

Life Expectancy with Depression_x_
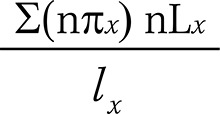


Healthy life expectancy (HLE) is the average number of years an individual is expected to live without depression, starting at an exact age x; life expectancy with depression represents the average number of years an individual is expected to live with depression, starting at an exact age x. To calculate HLE and life expectancy with depression, we used the prevalence of depression for individuals aged x to x + n (_n_π_x_) (obtained from the SABE survey), the total number of years of life expectancy for individuals aged x to x + n (_n_L_x_), and the survival probability for exact age x (l_x_). The last two variables were obtained from the life table generated from estimates provided by SEADE. Total life expectancy (TLE) is the sum of healthy (HLE) and unhealthy years of life. These estimates are independent of the age structure of the population.

The SABE survey was approved by the Research Ethics Committee of the Faculdade de Saúde Pública, Universidade de São Paulo, Brazil (Process: 67/99) and by the Brazilian National Ethics Committee (Process 315/99). Participation in the study was voluntary, and each respondent signed an informed consent form. The present study was approved by the Institutional Review Board at the University of Illinois.

## RESULTS

In 2000 and 2010, respectively, 281 (13.1%) and 65 subjects (4.8%) had missing data on depressive symptoms. In both years, those with missing data were older than those with complete data (in 2000, 80.8 years *versus* 72.1, p < 0.001; in 2010, 83.5 years *versus* 72.3, p < 0.001); however, there were no sex differences between the two groups.

Table 1 shows the results related to the prevalence of depression in 2000 and 2010 (weighted estimates) in the Sao Paulo population of older adults. Among the 1,862 participants in the 2000 sample, 21.8% (95%CI 19.7–24.0) were categorized as having depression (i.e., scored 6 or more on the GDS scale). The prevalence rate did not change over time: 21.1% (95%CI 18.8–23.6) of the 1,280 older adults in the 2010 sample were categorized as having depression. In the logistic regression models (results not shown, but available upon request), depression was more common among women than among men in both periods, even after controlling for age (p < 0.0001). The depression rate for men was 16.3% in 2000 and 14.4% in 2010, whereas the rate for women was about one fourth of the population in each year (25.7% in 2000 and 25.2% in 2010).

**Table 1:** Prevalence and 95%CI of depression among older adults in Sao Paulo, Brazil: 2000 and 2010 (weighted estimates).

Sex and age groups	2000		2010	
	
	%	95%CI	%	95%CI
All participants	21.8	19.7–24.0	21.1	18.8–23.6
60-64	21.4	17.7–25.7	23.3	18.9–28.3
65-69	24.5	20.3–29.2	15.1	10.8–20.6
70-74	21.3	17.0–26.4	20.6	15.5–26.9
75-79	18.7	15.0–23.1	23.7	17.3–31.5
80-84	16.9	12.4–22.7	22.8	16.3–30.8
≥ 85	23.0	15.1–33.4	22.0	16.0–29.5
Males	16.3	13.4–19.6	14.4	11.2–18.2
60-64	14.6	10.0–21.0	15.3	9.8–23.2
65-69	17.7	12.1–25.2	8.5	3.7–18.1
70-74	19.5	13.1–27.9	11.0	5.3–21.5
75-79	14.4	9.9–20.7	17.4	8.8–31.4
80-84	13.1	7.9–20.9	14.8	6.7–29.5
≥ 85	11.9	5.6–23.5	21.5	13.0–33.3
Females	25.7	22.9–28.7	25.2	22.1–28.5
60-64	26.8	21.5–32.8	28.6	22.7–35.4
65-69	29.5	23.9–35.9	19.0	13.2–26.4
70-74	22.5	17.1–29.2	25.9	19.1–34.2
75-79	21.4	16.3–27.5	27.1	19.0–37.1
80-84	18.9	12.8–26.9	26.3	18.2–36.4
≥ 85	29.7	18.3–44.5	22.5	14.8–32.8

TLE increased in Sao Paulo from 2000 to 2010 for both men and women (Table 2). TLE at age 60 increased from 17.5 years to 19.4 years for men and from 22.0 years to 23.7 years for women. The estimated average number of years lived without depression also increased. Among men, HLE at age 60 was 14.7 years in 2000 and 16.7 years in 2010, an increase of two years. Among women, the gain was smaller: HLE at age 60 was 16.5 years in 2000 and 17.8 years in 2010.

In both 2000 and 2010, the ratio of healthy years to total years was higher for men than for women. This result suggests that although women lived longer than men, on average, women were more likely to spend a higher percentage of their remaining lives with depression than men. Both men and women experienced very little change in life expectancy with depression over time. On average, women at age 60 were expected to live 5.5 years with depression in 2000 and 5.9 years in 2010. For men at age 60, life expectancy with depression was 2.8 years in 2000 and 2.7 years in 2010.

## DISCUSSION

Findings show that from 2000-2010, both TLE and life expectancy without depression increased among older adults in Sao Paulo, which may reflect living more years with higher quality of life, less disability, and better health status^28^. Given the association between depression and mortality, the recent gains in life expectancy without depression can increase TLE by reducing the number of premature deaths due to several causes (e.g., suicide).

Life expectancy and HLE are related to economic and social capital as well as social inequalities. In Brazil, the 2000-2010 period was marked by economic growth, improvements in social indicators, and reductions in social inequalities. According to the World Bank’s World Development Indicators database, *per capita* gross domestic product (measured in US dollars at 2005 prices) grew from US$4,407 in 2000 to US$5,618 in 2010^26^. The human development index, a composite measure including health, schooling, and income, has also improved in Brazil over recent decades^e^. The Gini coefficient, a measure of income inequality ranging from 0 (complete equality) to 1 (complete inequality), fell from 0.59 in 2000 to 0.54 in 2009. These values indicate a favorable change in several social and economic factors, which may have increased both TLE and life expectancy without depression.

The results support previous findings showing a higher prevalence of depression and depressive symptoms among older women than among older men in developing countries, including Brazil^3^^,^^4^^,^^20^^,^^32^. A larger proportion of older women than older men in Sao Paulo have depression and women can expect to live a higher proportion of their remaining lives with depression than men. These findings are consistent with previous research on life expectancy and HLE, which has found that even though women live longer lives, they live more years with health problems such as disability, cognitive impairment, and mental health problems^2^.

This study has some limitations. First, the measure of depression prevalence differed from the measure used in other studies. In this study, participants’ depressive symptoms were reported using the GDS 15-item subscale, and we used a cutoff score of 6. Other studies on the prevalence of depressive symptoms among Brazilians have used other measures, different cutoff points, or alternative ways of dealing with item nonresponse, all of which influence the estimated prevalence rate. For example, our estimate was higher than a previous estimate of the depression rate for older adults in Sao Paulo from 2002-2004 (22.0% *versus* 13.0%)^3^; which measured depression based on questions from the GDS-30 and the Center for Epidemiologic Studies Depression Scale (CES-D 10)^17^^,^^33^. The current results also differ from the original SABE reports^19^, which used different procedures for handling item nonresponse. Finally, our results were lower than the ones of a meta-analysis that reported a 26.0% prevalence of clinically significant depressive symptoms among older community-dwelling adults in Brazil^4^.

A second limitation of the study is related to the data collection procedures for depressive symptoms; these symptoms were self-reported by those who were cognitively intact, but those with cognitive limitations (n = 198 in 2000 and n = 196 in 2010) failed to provide information on depressive symptoms. Although missing data could also be considered an important limitation, when we compared the characteristics of respondents with missing data and those with no missing data (at baseline), only age differed significantly. In further analyses (results not shown, but available upon request), we imputed data for those with missing information on more than five items. Imputed prevalence rates differed slightly from the rates used in the study (21.2% *versus* 21.8% in 2000, respectively, and 21.7% *versus* 21.1% in 2010, respectively). When imputed values were calculated separately by sex, differences remained small, with all imputed prevalence rates decreasing slightly, except the rate for women in 2010 (imputed rate was 25.9%, compared with the 25.2% rate used in the analyses). Even when separated by sex, all imputed values were within the confidence intervals presented in Table 2.

**Table 2:** Total life expectancy (TLE) and life expectancy without depression (HLE) among older adults in Sao Paulo, Brazil: 2000 and 2010.

Sex and age	2000					2010				

	TLE	HLE	95%CI	% of remaining years without depression	Life expectancy with depression	TLE	HLE	95%CI	% of remaining years without depression	Life expectancy with depression
Total										
60	20.0	15.7	14.7–16.6	78.7	4.3	21.6	17.1	15.7–18.2	79.0	4.5
65	16.5	13.0	12.1–13.8	78.7	3.5	17.9	14.3	13.1–15.3	79.7	3.6
70	13.4	10.7	9.9–11.4	79.9	2.7	14.5	11.3	10.3–12.2	77.8	3.2
75	10.5	8.5	7.8–9.0	80.6	2.0	11.4	8.8	7.9–9.5	77.1	2.6
80	8.1	6.5	5.8–7.0	80.1	1.6	8.7	6.8	6.1–7.3	77.6	2.0
85	6.1	4.7	4.1–5.2	77.0	1.4	6.4	5.0	4.5–5.4	78.0	1.4
Males										
60	17.5	14.7	13.4–15.6	84.0	2.8	19.4	16.7	14.6–17.9	86.1	2.7
65	14.3	12.0	10.8–12.8	83.7	2.3	16.0	13.8	12.0–14.9	86.5	2.2
70	11.5	9.7	8.8–10.4	84.6	1.8	12.8	10.9	9.3–11.8	85.0	1.9
75	9.1	7.9	7.1–8.3	86.5	1.2	10.1	8.3	6.9–9.1	82.0	1.8
80	7.0	6.1	5.4–6.5	87.3	0.9	7.7	6.3	5.3–7.0	82.4	1.4
85	5.4	4.8	4.1–5.1	88.3	0.6	5.7	4.5	3.8–5.0	78.9	1.2
Females										
60	22.0	16.5	14.8–17.9	75.0	5.5	23.7	17.8	15.7–19.4	75.0	5.9
65	18.2	13.7	12.3–14.9	75.5	4.5	19.6	14.9	13.2–16.3	76.2	4.7
70	14.7	11.3	10.1–12.3	77.0	3.4	15.9	11.8	10.3–13.1	74.5	4.1
75	11.5	8.8	7.7–9.6	76.6	2.7	12.4	9.3	8.0–10.3	75.0	3.1
80	8.7	6.5	5.6–7.3	75.3	2.2	9.4	7.1	6.2–7.9	75.8	2.3
85	6.5	4.5	3.6–5.3	70.0	2.0	6.8	5.3	4.6–5.8	77.5	1.5

A third limitation of the study is that estimates of depression may be biased because we did not include respondents living in nursing homes at baseline and depression may be higher among this segment of the population. However, this population in Brazil is small – about 1.0% according to Camarano and Kanso^10^ and 0.5% according to the SABE survey; therefore, this bias is likely trivial.

Other limitations originate from the application of the Sullivan method. We employed the Sullivan method because the goal of the article was to evaluate trends over time in life expectancy with and without depression. However, we used a stationary population approach to interpret the estimates produced via Sullivan method, and thus results should be interpreted with caution because the data are dependent on past conditions of the population^22^. Another potential issue related to using mortality rates from life tables is that we did not formally account for the association between depression and increased mortality risk^25^. An alternative to the current analytical strategy would be to incorporate mortality by health state and health transitions (i.e., incidence and recovery) using multistate life tables^21^. However, as shown in other settings, longitudinal surveys tend to underestimate deaths as a result of the omission of respondents living in nursing homes in the baseline data as well as the potential of missing deaths in follow-up waves^18^^,^^29^. Finally, due to the lack of nationally representative data on the depression prevalence in Brazil, the results from this study reflect only the experiences of older adults in Brazil’s largest city, which may differ from the experiences of older people throughout the Country.

Life expectancy without depression increased from 2000 to 2010 in Sao Paulo. Further improvements in quality of life can be obtained by early diagnosis and treatment of individuals with depression. However, the underdiagnosis of depression is a serious health problem, particularly in countries such as Brazil in which many residents lack adequate access to healthcare services for diagnosis and treatment. In order to expand services and healthcare, the number of mental health care workers must increase, and access to care must improve.

Given the limited resources available, community and primary health workers can be trained to perform some roles under the supervision of specialists. Some authors have called for “task shifting” programs in which nonspecialists can complete certain tasks, such as identifying cases, providing referrals, supporting medication adherence, and delivering psychosocial treatment^13^^,^^15^. Given the associations between mental health conditions and other health conditions, integrating mental health care into general health care is another change that has the potential to improve outcomes^13^. In Brazil, the current *Política Nacional de Saúde do Idoso* (PNSI – National Policy for the Health of the Elderly) seeks to improve older adults’ health and well-being through improved immunization and distribution of medication^23^. A more diverse and larger workforce is also needed to deliver interventions aimed at modifying behaviors – such as physical exercise – that can improve the health outcomes of individuals with depressive symptoms^5^.

Finally, other innovations may involve using technology to improve medical care by expanding training and improving access to care and treatment in Brazil. Ultimately, empowering Brazilian individuals with depression and their families can lead to additional advocacy for better services, improved social inclusion, and reduced stigma^13^. All of these changes have the potential to facilitate early diagnosis, better treatment, and increased healthy life expectancy.

## Footnotes

a Comisión Económica para América Latina y el Caribe. Mortalidad. Observatório Demográfico. 2007;2(4). Available from: http://www.cepal.org/publicaciones/xml/9/33269/OD-4-contents-introduction.pdf

b Comisión Económica para América Latina y el Caribe. Proyecciones de población. Observatório Demográfico. 2012. Available from: http://repositorio.cepal.org/bitstream/handle/11362/7118/S2012922_mu.pdf?sequence=1

c Mortality data were obtained directly with Dr. Carlos Eugenio de Carvalho Ferreira, director of the Population Projection Area at SEADE Foundation.

d Jagger C, Hauet E, Brouard N. Health expectancy calculation by the Sullivan Method: a practical guide: European concerted action on the harmonization of health expectancy calculations in Europe (EURO-REVES). Paris; 2001 (REVES paper, nº 408).

e Klugman J. Human development report 2011: sustainability and equity: a better future for all. (November 2, 2011). UNDP-HDRO Human Development Reports, 2011. Available from: http://ssrn.com/abstract=2294671

## References

[1] Almeida OP, Almeida SA. Confiabilidade da versão brasileira da Escala de Depressão em Geriatria (GDS) versão reduzida. *Arq Neuropsiquiatr*. 1999;57(2B):421-6. DOI:10.1590/S0004-282X1999000300013 10.1590/s0004-282x1999000300013 10450349

[2] Andrade FC, Corona LP, Lebrão ML, Duarte YA. Life expectancy with and without cognitive impairment among Brazilian older adults. *Arch Gerontol Geriatr*. 2014;58(2):219-25. DOI:10.1016/j.archger.2013.10.007 10.1016/j.archger.2013.10.007 24246301

[3] Barcelos-Ferreira R, Pinto Junior JA, Nakano EY, Steffens DC, Litvoc J, Bottino CMC. Clinically significant depressive symptoms and associated factors in community elderly subjects from Sao Paulo, Brazil. *Am J Ger Psychiatry*. 2009;17(7):582-90. DOI:10.1097/JGP.0b013e3181a76ddc 10.1097/JGP.0b013e3181a76ddc 19546654

[4] Barcelos-Ferreira R, Izbicki R, Steffens DC, Bottino C. Depressive morbidity and gender in community-dwelling Brazilian elderly: systematic review and meta-analysis. *Int Psychogeriatr*. 2010;22(05):712-26. DOI:10.1017/S1041610210000463 10.1017/S1041610210000463 20478096

[5] Barcelos-Ferreira R, Yoshio Nakano E, Steffens DC, Bottino CMC. Quality of life and physical activity associated to lower prevalence of depression in community-dwelling elderly subjects from Sao Paulo. *J Affect Disord*. 2013;150(2):616-22. DOI:10.1016/j.jad.2013.02.024 10.1016/j.jad.2013.02.024 23499164

[6] .Barendregt JJ. Incidence- and prevalence-based SMPHs: making the twain meet. In: Murray CJL, Salomon JA, Mathers CD, Lopez AD, editors. Summary measures of population health: concepts, ethics, measurement and applications. Geneva: World Health Organization; 2002. p. 221-31.

[7] Beekman AT, Penninx BW, Deeg DJ, Beurs E, Geerlings SW, Tilburg W. The impact of depression on the well‐being, disability and use of services in older adults: a longitudinal perspective. *Acta Psychiatr Scand*. 2002;105(1):20-7. DOI:10.1034/j.1600-0447.2002.10078.x 10.1034/j.1600-0447.2002.10078.x 12086221

[8] Bhattarai N, Charlton J, Rudisill C, Gulliford MC. Prevalence of depression and utilization of health care in single and multiple morbidity: a population-based cohort study. *Psychol Med*. 2013;43(7):1423-31. DOI:10.1017/S0033291712002498 10.1017/S0033291712002498 23114010

[9] Bruce ML, Seeman TE, Merrill SS, Blazer DG. The impact of depressive symptomatology on physical disability: MacArthur studies of successful aging. *Am J Public Health*. 1994;84(11):1796-9. DOI:10.2105/AJPH.84.11.1796 10.2105/ajph.84.11.1796 PMC1615223 7977920

[10] Camarano AA, Kanso S. As instituições de longa permanência para idosos no Brasil. *Rev Bras Estud Popul*. 2010;27(1):232-5. DOI:10.1590/S0102-30982010000100014

[11] Castro-de-Araujo LFS, Barcelos-Ferreira R, Martins CB, Bottino C. Depressive morbidity among elderly individuals who are hospitalized, reside at long-term care facilities, and are under outpatient care in Brazil: a meta-analysis. *Rev Bras Psiquiatr*. 2013;35(2):201-7. DOI:10.1590/1516-4446-2012-0905 10.1590/1516-4446-2012-0905 23904028

[12] Chisholm D, Diehr P, Knapp M, Patrick D, Treglia M, Simon G et al. Depression status, medical comorbidity and resource costs: evidence from an international study of major depression in primary care (LIDO). *Br J Psychiatry*. 2003;183(2):121-31. DOI:10.1192/bjp.183.2.121 10.1192/bjp.183.2.121 12893665

[13] DeSilva M, Samele C, Saxena S, Patel V, Darzi A. Policy actions to achieve integrated community-based mental health services. *Health Aff (Millwood)*. 2014;33(9):1595-602. DOI:10.1377/hlthaff.2014.0365 10.1377/hlthaff.2014.0365 25201664

[14] DiMatteo MR, Lepper HS, Croghan TW. Depression is a risk factor for noncompliance with medical treatment: meta-analysis of the effects of anxiety and depression on patient adherence. *Arch Intern Med*. 2000;160(14):2101-7. DOI:10.1001/archinte.160.14.2101 10.1001/archinte.160.14.2101 10904452

[15] Fairburn CG, Patel V. The global dissemination of psychological treatments: a road map for research and practice. *Perspectives*. 2014;171(5):495-8. DOI:10.1176/appi.ajp.2013.13111546 10.1176/appi.ajp.2013.13111546 24788281

[16] Joffe H, Chang Y, Dhaliwal S, Hess R, Thurston R, Gold E et al. Lifetime history of depression and anxiety disorders as a predictor of quality of life in midlife women in the absence of current illness episodes. *Arch Gen Psychiatry*. 2012;69(5):484-92. DOI:10.1001/archgenpsychiatry.2011.1578 10.1001/archgenpsychiatry.2011.1578 PMC3584338 22566580

[17] Kohout FJ, Berkman LF, Evans DA, Cornoni-Huntley J. Two shorter forms of the CES-D depression symptoms index. *J Aging Health*. 1993: 5:179-93. DOI: 10.1177/089826439300500202 10.1177/089826439300500202 10125443

[18] Kristman V, Manno M, Côté P. Loss to follow-up in cohort studies: how much is too much? *Eur J Epidemiol*. 2004;19(8):751-60. DOI:10.1023/B:EJEP.0000036568.02655.f8 10.1023/b:ejep.0000036568.02655.f8 15469032

[19] Lebrão ML, Laurenti R. Saúde, bem-estar e envelhecimento: o estudo SABE no município de São Paulo. *Rev Bras Epidemiol*. 2005;8(2):127-41. DOI:10.1590/S1415-790X2005000200005

[20] Li D, Zhang D, Shao J, Qi X, Tian L. A meta-analysis of the prevalence of depressive symptoms in Chinese older adults. *Arch Gerontol Geriatr*. 2014;58(1):1-9. DOI:10.1016/j.archger.2013.07.016 10.1016/j.archger.2013.07.016 24001674

[21] Lievre A, Alley D, Crimmins EM. Educational differentials in life expectancy with cognitive impairment among the elderly in the United States. *J Aging Health*. 2008;20(4):456-77. DOI:10.1177/0898264308315857 10.1177/0898264308315857 PMC2966893 18448687

[22] .Mathers C. Health expectancies: an overview and critical appraisal. In: Murray C, Salomon JA, Mathers CD, Lopez AD, editors. Summary measures of population health: concepts, ethics, measurement and applications. Geneva: World Health Organization; 2002. p.177-204.

[23] Miyata DF, Vagetti GC, Fanhani HR, Pereira JG, Andrade OG. Políticas e programas na atenção à saúde do idoso: um panorama nacional. *Arq Cienc Saude Unipar*. 2005;9(2):135-40.

[24] Ownby RL, Crocco E, Acevedo A, John V, Loewenstein D. Depression and risk for Alzheimer disease: systematic review, meta-analysis, and metaregression analysis. *Arch Gen Psychiatry*. 2006;63(5):530-8. DOI:10.1001/archpsyc.63.5.530 10.1001/archpsyc.63.5.530 PMC3530614 16651510

[25] Pan A, Sun Q, Okereke OI, Rexrode KM, Hu FB. Depression and risk of stroke morbidity and mortality: a meta-analysis and systematic review. *JAMA*. 2011;306(11):1241-9. DOI:10.1001/jama.2011.1282 10.1001/jama.2011.1282 PMC3242806 21934057

[26] Pao H, Fu H. Renewable energy, non-renewable energy and economic growth in Brazil. *Renew Sustain Energy Rev*. 2013;25:381-92. DOI:10.1016/j.rser.2013.05.004

[27] Penninx BW, Leveille S, Ferrucci L, Eijk JT, Guralnik JM. Exploring the effect of depression on physical disability: longitudinal evidence from the established populations for epidemiologic studies of the elderly. *Am J Public Health*. 1999;89(9):1346-52. DOI:10.2105/AJPH.89.9.1346 10.2105/ajph.89.9.1346 PMC1508750 10474551

[28] Reynolds SL, Haley WE, Kozlenko N. The impact of depressive symptoms and chronic diseases on active life expectancy in older Americans. *Am J Geriatr Psychiatry*. 2008;16(5):425-32. DOI:10.1097/JGP.0b013e31816ff32e 10.1097/JGP.0b013e31816ff32e 18448853

[29] Romero-Ortuno R, Fouweather T, Jagger C. Cross-national disparities in sex differences in life expectancy with and without frailty. *Age Ageing*. 2014;43(2):222-8. DOI:10.1093/ageing/aft115 10.1093/ageing/aft115 PMC3927770 23917483

[30] Wancata J, Alexandrowicz R, Marquart B, Weiss M, Friedrich F. The criterion validity of the Geriatric Depression Scale: a systematic review. *Acta Psychiatr Scand*. 2006;114(6):398-410. DOI:10.1111/j.1600-0447.2006.00888.x 10.1111/j.1600-0447.2006.00888.x 17087788

[31] Win S, Parakh K, Eze-Nliam CM, Gottdiener JS, Kop WJ, Ziegelstein RC. Depressive symptoms, physical inactivity and risk of cardiovascular mortality in older adults: the Cardiovascular Health Study. *Heart*. 2011;97(6):500-5. DOI:10.1136/hrt.2010.209767 10.1136/hrt.2010.209767 PMC3044493 21339320

[32] Yaka E, Keskinoglu P, Ucku R, Yener GG, Tunca Z. Prevalence and risk factors of depression among community dwelling elderly. *Arch Gerontol Geriatr*. 2014;59(1):150-4. DOI:10.1016/j.archger.2014.03.014 10.1016/j.archger.2014.03.014 24767692

[33] Yesavage JA, Brink T, Rose TL, Lum O, Huang V, Adey M et al. Development and validation of a geriatric depression screening scale: a preliminary report. *J Psychiatr Res*. 1982;17(1):37-49. DOI:10.1016/0022-3956(82)90033-4 10.1016/0022-3956(82)90033-4 7183759

[34] Zvolensky MJ, Bakhshaie J, Sheffer C, Perez A, Goodwin RD. Major depressive disorder and smoking relapse among adults in the United States: A 10-year, prospective investigation. *Psychiatry Res*. 2015;226(1):73-7. DOI:10.1016/j.psychres.2014.11.064 10.1016/j.psychres.2014.11.064 PMC4448723 25650047

